# Reciprocal Crosstalk between Dendritic Cells and Natural Killer T Cells: Mechanisms and Therapeutic Potential

**DOI:** 10.3389/fimmu.2017.00570

**Published:** 2017-05-24

**Authors:** Christian W. Keller, Stefan Freigang, Jan D. Lünemann

**Affiliations:** ^1^Institute of Experimental Immunology, Laboratory of Neuroinflammation, University of Zurich, Zurich, Switzerland; ^2^Institute of Pathology, Laboratory of Immunopathology, University of Bern, Bern, Switzerland; ^3^Department of Neurology, University Hospital Zurich, Zurich, Switzerland

**Keywords:** dendritic cells, natural killer T cells, CD1d, glycolipids, immunotherapy

## Abstract

Natural killer T cells carrying a highly conserved, semi-invariant T cell receptor (TCR) [invariant natural killer T (iNKT) cells] are a subset of unconventional T lymphocytes that recognize glycolipids presented by CD1d molecules. Although CD1d is expressed on a variety of hematopoietic and non-hematopoietic cells, dendritic cells (DCs) are key presenters of glycolipid antigen *in vivo*. When stimulated through their TCR, iNKT cells rapidly secrete copious amounts of cytokines and induce maturation of DCs, thereby facilitating coordinated stimulation of innate and adaptive immune responses. The bidirectional crosstalk between DCs and iNKT cells determines the functional outcome of iNKT cell-targeted responses and iNKT cell agonists are used and currently being evaluated as adjuvants to enhance the efficacy of antitumor immunotherapy. This review illustrates mechanistic underpinnings of reciprocal DCs and iNKT cell interactions and discusses how those can be harnessed for cancer therapy.

## Natural Killer T Cells (NKT Cells)

Natural killer T cells belong to the group of innate-like T lymphocytes and represent an important link between the innate and the adaptive immune response. They can be activated in both antigen-dependent and independent manners, secrete large amounts of cytokines upon activation, and exhibit remarkable functional plasticity with both pro-inflammatory and immunoregulatory characteristics (1, 2). Depending on their T cell receptor (TCR), CD1d-restricted NKT cells are subdivided into type I or invariant NKT (iNKT) cells, and type II or diverse NKT (dNKT) cells. Herein, we will focus on the unique iNKT cell subset, which expresses a semi-invariant TCR and we refer the reader to excellent reviews on type II NKT cells elsewhere (3, 4).

In 1986 and 1987, respectively, three key discoveries facilitated the identification of this innate-like T cell subset. Two groups independently described a Vβ8-overexpressing, double-negative thymocyte subset in mice, while a third research team cloned an invariant TCR Vα14-Jα18 rearrangement from a set of murine suppressor T cell hybridomas (5–7). It was not until 10 years later that the ligands, which these peculiar cells recognize, were identified (8).

Type I NKT cells are characterized by the expression of a semi-invariant TCR (Vα14Jα18 paired with Vβ8, Vβ7, or Vβ2 in mice and Vα24Jα18/Vβ11 in humans) (3, 9). Interestingly, the Vα14 TCR is exclusively used by iNKT cells but not by conventional T cells (10). Furthermore, iNKT cell subsets bear morphological markers on their surface that were believed to be characteristic for natural killer (NK) cells like NKG2D (11), KLRG (12), IL-12 receptor (13), or NK1.1 (CD161) (8, 14–16). However, although expression of these molecules may characterize some NKT cell subsets, other subsets do not share these NK cell markers. Therefore, the more stringent characteristic of NKT cells appears to be their CD1d restriction (1).

Unlike conventional CD4^+^ or CD8^+^ T cells, iNKT cells recognize antigenic glycolipids presented *via* the monomorphic MHC class I-like molecule CD1d (17, 18). iNKT cell responses have proven to be highly conserved between humans and mice. They enhance the activation of innate immune cells, such as dendritic cells (DCs) and NK cells, and shape immune responses in concert with other lymphocytes, such as B cells. Thereby, iNKT cells not only act as an amplification relay but bridge innate and adaptive immunity (19–22). The frequencies of iNKT cells among total lymphocytes differ greatly between tissues and the possibility of detecting these unconventional T cells has greatly improved by the introduction of lipid-loaded CD1d-tetramers (15, 16). In mice, iNKT cells are most abundant in the liver (10–30%) and the spleen (0.5–1.5%) with lower frequencies found in thymus, blood, bone marrow (all 0.2–0.5%), and lymph nodes (0.1–0.2%). In humans, substantial interindividual variability is observed. However, high iNKT cell frequencies are detected in the liver (1%), omentum (10%), the adipose tissue (in which iNKT cell frequencies vary between 0.5 and 1% of total CD3^+^ cells) (23), and in healthy donors iNKT cells represent 0.01–0.5% of PBMCs (24, 25). The iNKT cell subset develops in the thymus, emerges from the same progenitor pool as conventional T cells, and undergoes somatic recombination and thymic selection. Rather than *via* thymic epithelial cells, iNKT cells are positively selected through interaction with double-positive thymocytes that CD1d-present endogenous ligands, leading to an unusually strong TCR signal. The directing of iNKT cell precursors toward a particular subset lineage may involve specific endogenous selecting lipid antigens (19, 26, 27). The majority of human thymic iNKT cells egresses during early fetal development and CD4^+^CD8^−^ iNKT cells are already present at birth, whereas murine iNKT cells only emerge during the first postnatal week (25, 28, 29). Distinct human iNKT cell subsets include CD4^+^/CD8^−^, CD4^−^/CD8^−^, and CD4^−^/CD8^+^ whereas in mouse CD4^+^/CD8^−^ and CD4^−^/CD8^−^ subsets prevail (25).

## iNKT Cell Heterogeneity and Effector Functions

Initially believed to be a rather rigid and homogenous cell population that merely acts upon TCR stimulation, it became recently clear that based on their respective transcriptional programs, distinct iNKT cell subsets with designated functional properties exist and that iNKT cells may balance immune homeostasis *via* their steady-state activity. TCR-induced transcription factors Egr2 and Egr1 lead to transcription of PLZF, the key transcriptional factor during the development of iNKT cells (30). In fact, although only a subset of fully matured iNKT cells are positive for PLZF, the majority of iNKT cells expresses this transcription factor at one point during development (31–34). Depending on the subsequent transcriptional program, thymic CD24^hi^/CD69^+^ iNKT cell precursors diverge into distinct sublineages (35). T_H_1 iNKT cells (NKT1) express T-bet and Bhlhe40 and mainly release IFNγ upon TCR ligation. T_H_2 iNKT cells (NKT2) predominantly express GATA3 and PLZF and release IL-4 and IL-13 already in steady state. IL-17-producing iNKT17 express RORγt, a subset of Bcl-6-dependent, CXCR5- and PD1-expressing iNKT follicular helper cells secrete IL-21, thereby shaping B cell responses. IL-10-producing immunoregulatory NKT10 are FOXP3-negative but positive for the transcription factor E4BP4 (20, 27, 36–38). Recently, a KLRG-expressing subset of iNKT cells has been described, which shows an effector-memory-like phenotype and is able to mount stronger secondary responses to cognate antigen (12).

Invariant NKT cells can be activated either upon stimulation of their TCR by CD1d-presented glycolipid antigens, or in a TCR-independent manner (e.g., by cytokines) (39, 40). Upon activation, iNKT cells readily proliferate and undergo significant remodeling of their surface expression patterns with regards to several markers, such as NK1.1 and the semi-invariant TCR (41).

Although iNKT cells have adaptive characteristics, they exist in a preactivated memory-like effector state primed to release large amounts of immunomodulatory cytokines (including IFNγ, IL-4, IL-13, IL-17, GM-CSF, and TNF-α) not only upon engagement of their TCR but also in response to innate signals (13). One of their key features is the cytokine-mediated transactivation of other innate and innate-like immune cell subsets, thereby amplifying initial responses (19, 42–45). In addition, iNKT cells may also provide both antigen-specific cognate and non-cognate help for B cells (20, 46, 47) and in turn can be activated by B cells (48, 49). Interestingly, unlike the non-cognate iNKT cell–B cell interactions, antigen-specific iNKT cell help induces a more innate-biased B cell response, which is characterized by a discontinuous germinal center B cell expansion and rapid initial proliferation of IL-10-producing B cells, but fails to induce humoral memory (50).

A key difference between iNKT cells and conventional T cells are the kinetics of their responses, which in case of iNKT cells occur already within hours after engagement, as opposed to several days in the case of conventional T cells (1, 51). In line with this, iNKT cells have been reported to carry preformed mRNA of cytokines in their cytoplasm, which enables them to rapidly release large quantities of these effector molecules upon TCR ligation (52, 53). The translational regulation of preformed cytokine mRNA has been shown to be dependent on p38 MAPK (54).

Aside from rapidly releasing numerous immunomodulatory cytokines, iNKT cells also have immediate cytotoxic capacity, which correlates with the amount of surface CD1d on the target cell (55). While reports in patients suffering from acute myeloid leukemia (AML) and juvenile myelomonocytic leukemia describe (analogous to NK cell-mediated cytotoxicity) predominant usage of the perforin/granzyme B pathway in executing cytotoxicity, other reports in C57BL/6 mice ascribe a higher importance to Fas/FasL interaction (55). In addition to exerting direct effector functions, it becomes more and more apparent that iNKT may shape immune responses indirectly through crosstalk with other immune subsets.

Myeloid-derived suppressor cells (MDSCs) are a unique Gr1^+^ population of activated myeloid cells that retain an immature phenotype and are functionally able to dampen adaptive immune responses during malignancies and infection (56). De Santo et al. described an intriguing mechanism through which iNKT cells reverse the suppressive properties of MDSCs during influenza A virus (IAV) infection in a CD1d- and CD40:CD40L-dependent manner (57). While infection of both CD1d^−/−^ and Jα18^−/−^ mice with the IAV strain A/Puerto Rico/8/34 (PR8) lead to a more severe phenotype and a greater expansion of CD1d- and CD40-expressing MDSCs in the lungs of Jα18^−/−^ and CD1d^−/−^ mice as compared to PR8-infected wild-type mice, only adoptive transfer of iNKT cells into Jα18^−/−^ mice ameliorated the disease course and reduced MDSC numbers whereas CD1d^−/−^ mice remained hypersusceptible and depicted unchanged numbers of MDSCs. MDSCs isolated from the lungs of PR8-infected Jα18^−/−^ mice depicted a stronger suppressive activity as those from wild-type mice. Pulsing MDSCs with αGalCer or TLR agonists (for TLRs 3, 7/8, and 9) in the presence of iNKT cells reduced suppressive activity of MDSCs. The results of this study suggest that TLR-mediated upregulation of (yet to be defined) endogenous iNKT cell ligands contribute to the iNKT cell-mediated modulation of MDSC suppressive activity during IAV infection. Accordingly, immunosuppressive properties of MDSCs isolated from IAV-infected patients could be reversed by iNKT cells (57). Likewise, it was shown that MDSCs pulsed with tumor-associated antigens and the prototypic iNKT cell agonist αGalCer fail to suppress cytotoxic T lymphocytes (CTLs) and do not induce generation of FOXP3^+^ T regulatory cells (TREGs), thus leading to longer survival of mice in a murine metastatic tumor model. Activated iNKT cells are able to modify MDSCs, transforming them back to a more immunogenic APC phenotype (58). MDSCs do not only include macrophages but also neutrophils, which acquire immunosuppressive properties such as IL-10-secretion, resulting in damping of antigen-specific T cell responses (59). It was shown that the acute-phase protein serum amyloid A 1 fosters iNKT-mediated conversion of suppressive activity of neutrophils. This immunomodulatory crosstalk between iNKT cells and neutrophils is highly dependent on CD1d:TCR interaction (60). All-*trans*-retinoic acid (ATRA) is known to promote MDSC differentiation (61, 62). Exposure of αGalCer-loaded MDSCs with ATRA has shown to restore immunogenicity of this immune subset in an iNKT cell-dependent way (63). These findings extend the previously reported arsenal of iNKT cells to execute their immunomodulatory functions.

Like MDSCs, tumor-associated monocytes/macrophages (TAMs) are part of the tumor microenvironment but unlike MDSCs, TAMs are Gr1^−^ (64). Primary human neuroblastoma cells are CD1d^−^, however, the tumor neuroblastoma microenvironment is highly enriched for CD68^+^/CD1d^+^ TAMs, which aliment tumor growth mainly through secretion of IL-6. CD1d-dependent killing of growth-promoting TAMs *via* iNKT cells decelerated tumor growth in a NOD/SCID human neuroblastoma xenograft model (65). Tumor necrosis factor related apoptosis inducing ligand (TRAIL)-expressing autologous or allogeneic CD4^+^ iNKT cells induce apoptosis in myeloid leukemia cells derived from AML patients. However, TCR:CD1d interaction was not required for this effector function (66). Further TCR-independent effector functions include NKG2D-dependent cytotoxic degranulation (11), differential cytokine expression pattern upon stimulation with IL-2, IL-12, IL-18 (67) and potentiating NK-cell mediated cytotoxicity in an IL-2-dependent manner (68).

## iNKT Cell Activation by DCs

The CD1 family is comprised of five isoforms that can be partitioned in two groups. Group 1 consists of CD1a, CD1b, CD1c, and CD1e and group 2 only includes CD1d. While all isoforms can be found in humans, only CD1d is expressed in mice (69).

**Figure 1:**
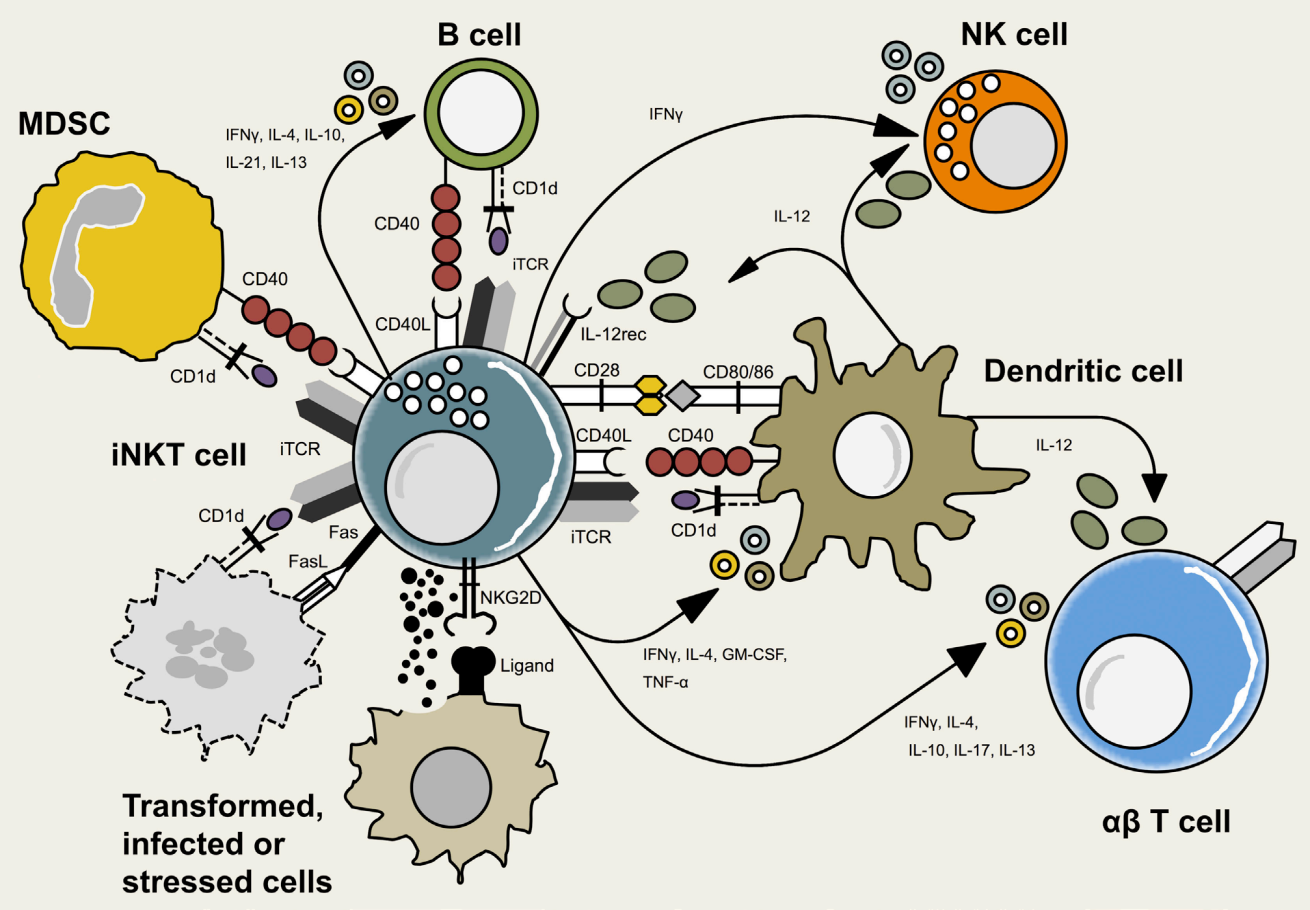
**Interactions between invariant natural killer T (iNKT) cells and other immune cells**. iNKT cells crosstalk with adaptive and innate immune cells including dendritic cells (DCs), B cells, natural killer (NK) cells, myeloid-derived suppressor cells (MDSCs), and conventional T cells. This reciprocal interaction is mediated *via* T cell receptor (TCR)-CD1d-recognition, interaction of co-stimulatory molecules or cytokines. Additionally, iNKT cells may interact with non-hematopoietic cells that expose signs of infection, transformation, or cell stress.

Dendritic cells constitutively express CD1d and may activate iNKT cells by presenting antigenic glycolipids. CD1d is a highly conserved non-polymorphic MHC class I-like transmembrane molecule; its expression is regulated by cytokines as well as through engagement of innate receptors (70). Similar to the structurally related MHC class I molecules, CD1d represents a heterodimer comprised of the CD1d heavy chain non-covalently coupled to β2-microglobulin. Many hematopoietic and non-hematopoietic cell types express CD1d on their surface either constitutively or upon activation (71–75). However, in mice, constitutively CD1d-expressing DCs appear to be the most potent APCs for exogenous glycolipids (76–78). The interaction between iNKT cells and DCs is not unidirectional but characterized by reciprocal feedback loops depending on the chemical structure of the CD1d ligand as well as the nature of the APC (Figure 1) (9, 76). DCs acquire and CD1d present exogenous lipid antigens for the direct recognition by iNKT cells but may also transduce innate signals toward to the induction of iNKT cell responses (39, 40, 79). In many cases, the activation of iNKT cells results from a combination of TCR-mediated recognition of cognate lipid antigen and TCR-independent signals. For example, pattern recognition receptor (PRR)-bearing DCs will CD1d-present endogenous glycolipids in response to stimulation with pathogen-associated molecular patterns (39, 79–81). Recent studies suggest that PRR activation may specifically modulate the lysosomal processing of glycolipids in APCs to increase the abundance of endogenous iNKT cell agonists (80, 81). In concert with signals provided by pro-inflammatory cytokines secreted by PRR-activated DCs, the weaker TCR recognition of endogenous antigens is sufficient for iNKT cell activation (39, 40). To which extent similar cytokine signals are required for iNKT activation by microbes expressing stimulatory lipid antigens remains debated (13, 82). iNKT cells constitutively express the IL-12 receptor and PRR-mediated secretion of IL-12 by DCs triggers Stat4 phosphorylation and consecutive IFNγ secretion in iNKT cells (13, 83). Furthermore, direct cellular contact between DCs and iNKT cells in a CD40:CD40L-dependent manner provides a strong feed-forward signal, resulting in additional IL-12 production by DCs and consecutive further upregulation of the IL-12 receptor on iNKT cells. CD40/CD40L as well as CD28:CD80/CD86 interactions are required for subsequent iNKT cell-mediated-IFNγ secretion whereas IL-4-secretion was described to be solely dependent on CD28:CD80/CD86 interaction (83, 84). Co-administration of iNKT cell agonist αGalCer and OVA in CD40^−/−^ and CD40L^−/−^ mice leads to abrogation of CD4^+^ and CD8^+^ T cell responses, while DCs are not affected in their ability to present antigen on MHC class I or II and are capable of upregulating CD80/86 (77). Recent reports show that artificial APCs loaded with iNKT cell agonists can activate and expand human iNKT cells *in vitro* as potently as autologous immature DCs. Engineering artificial APCs with differential association to co-stimulatory factors will help to obtain valuable insights into the crosstalk between iNKT cells and DCs and will foster our understanding of how to harness their therapeutic potential (85–87).

## CD1d Trafficking in Mouse Professional APCs

**Figure 2:**
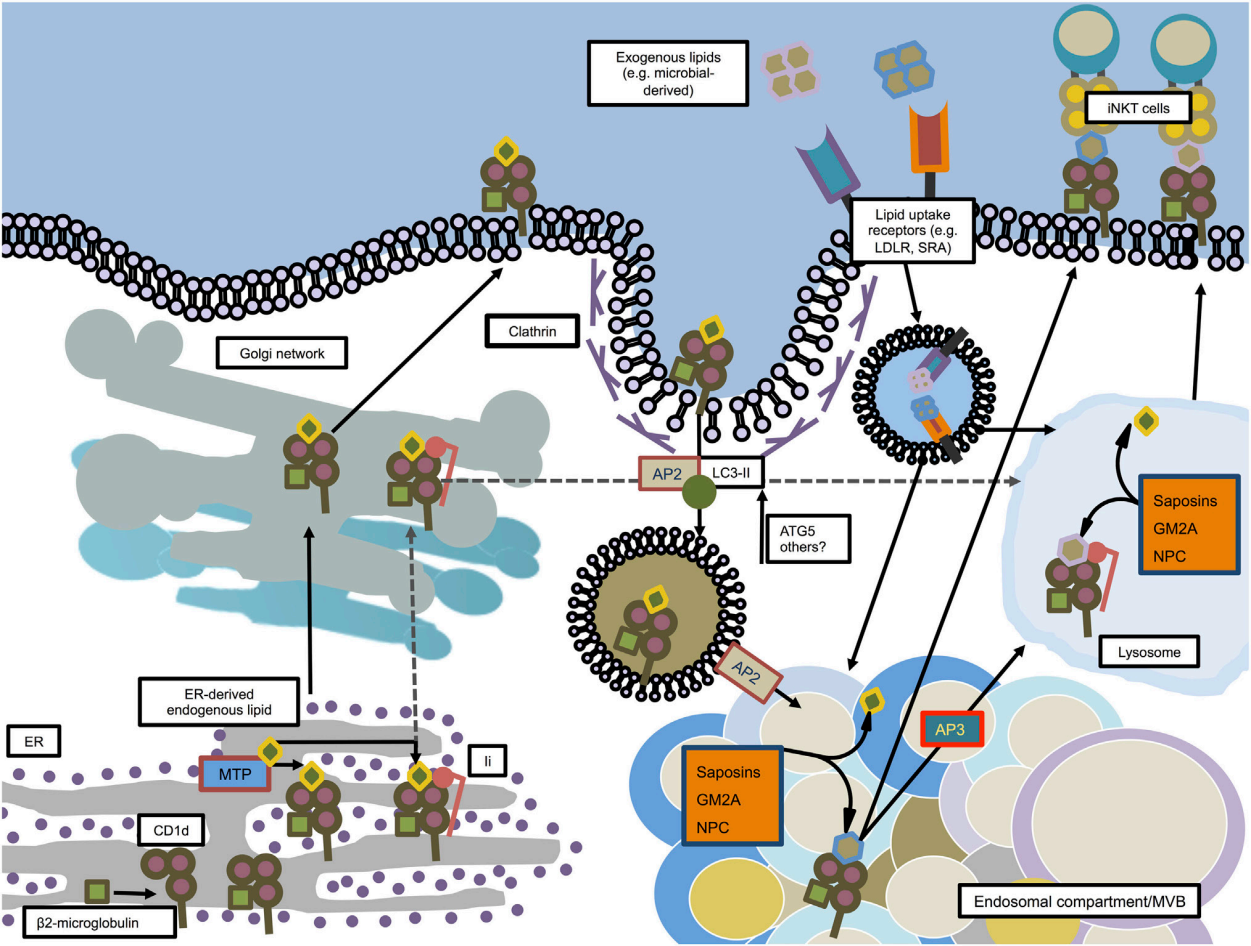
**CD1d trafficking and loading in mouse dendritic cells**. CD1d molecules are synthesized in the ER where they associate with β2-microglobulin. Microsomal triglyceride transfer protein (MTP) facilitates loading of ER-derived endogenous lipids onto CD1d in order to stabilize the molecule for further transport. In an independent pathway, some CD1d molecules associate in the ER with invariant chain (Ii). The Ii/CD1d complexes, after traveling through the *trans*-Golgi network, are directly guided to the lysosome. The non-Ii-associated CD1d molecules also pass the *trans*-Golgi network on their way to the plasma membrane. From there, facilitated by AP2 and members of the autophagy machinery, CD1d is internalized by clathrin-mediated endocytosis and guided toward endosomal compartments, where saposins, GM2 ganglioside activator (GM2A), and Niemann–Pick type C1 and C2 proteins (NPC1, NPC2) help exchanging endogenous lipids with exogenous or other endogenous lipids. From there, loaded CD1d may either be transported to the lysosome in an adaptor protein 3 (AP3)-dependent manner, or directly transported to the plasma membrane in order to interact with iNKT cells.

Similar to MHC class I molecules, CD1d molecules are synthesized, folded, and equipped with β2-microglobulin in the ER (Figure 2) (88). Analogous to the placeholder function of the pseudopeptide CLIP in MHC class II, CD1d most likely leaves the ER with an endogenous lipid in its antigen-binding groove in order to maintain stability. The biochemical nature of these lipids and the exact mechanisms underlying the respective transfer processes are not fully elucidated yet. However, the ER chaperone protein microsomal triglyceride transfer protein (MTP) has been suggested to load phospholipids onto nascent CD1d (89). CD1d, after having passed several protein quality control checkpoints, follows the secretory pathway and is being guided to the Golgi apparatus and subsequently reaches the cell surface (88, 90). From there, CD1d is being internalized in clathrin-coated pits *via* the interaction of the adaptor protein complex 2 (AP2) and adaptor protein 3 (AP3) through tyrosine-based sorting motifs present in the cytoplasmic tail of CD1d, and subsequently delivered to endosomal compartments (91–93). The autophagic machinery assists in the recruitment of AP2 to CD1d molecules. Loss of the essential autophagy protein ATG5 in DCs impaired clathrin-dependent internalization of CD1d molecules *via* AP2 and, thus, increased surface expression of stimulatory CD1d:glycolipid complexes, which resulted in enhanced iNKT activation (94).

Having generated a knock-in mouse by homologous recombination in which all CD1d is expressed as CD1d-EYFP, Sillé and colleagues described that in order to activate iNKT cells, endosomal sorting of CD1d is dependent on both its tyrosine-based sorting motif and on the association with the invariant chain (Ii) in peripheral DCs (95).

CD1d itself is unable to extract and acquire lipids from membranes and, therefore, is in need of lipid transfer proteins (LTPs) that, in analogy to the MHC class II/H2-DM interaction, facilitate loading of antigens within the endosomal/lysosomal compartment. Low molecular-weight proteins called saposins (A, B, C, and D), GM2 ganglioside activator (GM2A), and the Niemann–Pick type C1 and C2 proteins (NPC1 and NPC2, respectively) have so far been identified to mediate the loading of lipids on CD1d molecules (96–99). Additionally, in the cases of saposins and GM2A, it was reported that these molecules also aid in the unloading of lipids from the CD1d antigen-binding groove (93). Following sampling of antigens in the endo/lysosomal system, loaded CD1d molecules are being recycled to the cell surface, similar to MHC class II molecules, and present bound antigens for iNKT cell activation.

## Endogenous and Exogenous iNKT Cell Antigens

Different from MHC class I or II-restricted antigen presentation during which processing of proteins or larger peptides results in smaller antigenic peptides presented by polymorphic antigen-presenting molecules, monomorphic CD1d presents mainly unprocessed lipids of varying size and biochemical structure (100). Lipid antigens that stimulate CD1d-restricted iNKT cells comprise endogenous (also called self-lipids) and exogenous (e.g., microbial-derived) non-peptidic molecules. Within the mammalian class, self-lipids predominantly consist of glycosphingolipids (GSLs) and phosphoglycerolipids (69). Endogenous self-lipids have been described to be crucial for thymic selection of iNKT cells *via* CD4^+^CD8^+^ thymocytes (28, 98) but might also be involved in modulating antiviral and antineoplastic iNKT activity in the periphery (57, 79, 101). With regards to MHC molecules, methodological advances in proteomics allowed for progressive elucidation of the MHC-binding immunopeptidome in recent decades (102). In stark contrast, little is known about the endogenous lipid repertoire bound on CD1d *in vivo*, which is largely due to the fact that current techniques to extract CD1d molecules from cell membranes irretrievably entail the dissociation of CD1d-associated ligands. Although generation of secreted human CD1d molecules (sCD1d) by truncating the transmembrane and cytoplasmic domains shed some light on which lipid antigens are associated with CD1d (103), this approach implicates obvious shortcomings. Since the intracellular cytoplasmic tail of CD1d is required for trafficking of CD1d through endolysosomal compartments in which lipid exchange and transfer occurs (28, 104), the detected lipidom is unlikely to reflect the *in vivo* setting. Therefore, the development of more refined techniques is required for the unequivocal identification of *in vivo*-relevant endogenous CD1d ligands.

The lysosomal glycosphingolipid isoglobotrihexosylceramide (iGb3), a moderate activator of iNKT cells, has been proposed to function as a self-lipid (105). However, the biological relevance of this finding warrants further investigation (106, 107). Other candidate self-lipids to be involved in iNKT cell development are the peroxisomal-derived ether-bonded phospholipids 1-*O*-1′-(Z)-hexadecenyl-2-hydroxy-*sn*-glycero-3-phosphoethanolamine and 1-*O*-1′, 9′-(Z,Z)-octadecadienyl-2-hydroxy-*sn*-glycero-3-phosphoethanolamine. Not only depicted the synthetic plasmalogen C16-lysophosphatidylethanolamine (pLPE) similar iNKT cell stimulatory capacities as the prototypical agonist αGalCer but mice deficient in glyceronephosphate O-acyltransferase (GNPAT), the peroxisomal enzyme essential for synthesis of ether lipids, showed impaired iNKT cell development. However, GNPAT^−/−^ mice still harbored around 50% of iNKT cells found in GNPAT-competent mice (108). Other endogenous lipid antigens might, therefore, be involved in thymic selection of iNKT cells as well and the understanding of the relative contribution and distinct functions of a given endogenous CD1d ligand to iNKT cell biology will need further clarification.

Most exogenous CD1d ligands identified to date are of bacterial origin. iNKT cell-activating lipid antigens have been found in *Borrelia burgdorferi* [α-galactosyldiacylglycerols (αGalDAGs)] (109), *Sphingomonas* spp. (α-glucuronosylceramides and α–galacturonosylceramides) (40, 110, 111), *Streptococcus pneumoniae*, and group B *Streptococcus* [α-glucosyldiacylglycerols (αGlcDAGs)] (112), *Mycobacterium tuberculosis* (phosphatidylinnositol mannosides) (113), *Helicobacter pylori* (cholesteryl α-glucoside) (114), and *Bacteroides fragilis* (α-galactosylceramides) (115). But also the porifera *Agelas mauritianus* (α-galactosylceramides) (8, 116) and the ascomycete *Aspergillus fumigatus* (asperamide B) (117) have been reported to contain antigenic lipids that activate iNKT cells.

The α-linked monoglycosylceramide αGalCer, initially isolated from *Agelas mauritianus* was the first glycolipid identified to activate iNKT cells. Its synthetic derivative KRN7000 has become a commonly used experimental tool in iNKT cell research and to this day remains to be the most potent iNKT cell agonist (8, 116). Until recently, it was believed that mammalian cells are incapable of generating α-anomeric GSLs such as αGalCer. Making use of high-sensitivity biological assays, lipid immunopurification, and multiple reaction monitoring-mass spectrometry, Kain et al. reported that trace amounts of α-linked GSLs (both, αGluCer and αGalCer) are produced in mammalian cells and most likely function as endogenous ligands during thymic selection of iNKT cells (81).

## iNKT Cell-Mediated Maturation and Licensing of DCs

As a feedback loop, αGalCer-activated iNKT cells contribute to maturation of DCs *in vivo* resulting in increased cell surface expression of MHC class II, the co-stimulatory molecules CD40, CD80, CD86, and the endocytic receptor DEC-205. iNKT cell-matured DCs elicit specific CD4^+^ and CD8^+^ T cell responses against a co-administered peptide. The observed DC maturation is highly dependent on iNKT cells since administration of αGalCer fails to induce DC maturation in Jα18^−/−^ mice lacking iNKT cells (118). Challenge with OVA-expressing tumors demonstrated significant tumor resistance in animals that had been previously immunized with OVA in combination with the iNKT cell agonist αGalCer (119). In mice, the unique subset of CD8α^+^ DCs is able to cross-present extracellular antigens *via* MHC class I to evoke CTL responses (120). Both mouse (121) and human (122) studies have shown that cross-presentation is CD4^+^ T helper (T_H_) cell dependent. The interaction between mouse CD4^+^ T_H_ cells and DCs leads to release of CCL3 and CCL4 attracting CCR5-expressing CTLs to the site of cross-presentation (123). However, the CCR4-CCL17-dependent licensing of DCs by iNKT cells for cross-presentation has been described as an alternative pathway. iNKT cell-mediated upregulation of CCL17 in DCs required CD1d and spatial interaction between iNKT cells and DCs (124). Interestingly, Arora et al. reported that despite numerous cell types expressing high levels of CD1d, the CD8α^+^ DCs are the most competent presenters of lipid antigens *in vivo* (125). Whether and to which extent these mechanisms are translated to humans, remains to be addressed.

## Functional Outcomes of iNKT Cell Activation

Recognition of CD1d:glycolipid complexes *via* the iNKT cell TCR can result in either pro-inflammatory T_H_1-biased or T_H_2-biased cytokine production by iNKT cells (2, 18). Mechanisms that mediate such potentially opposing functional outcomes need to be taken into account in designing iNKT cell-targeting therapies. Differential expression of co-stimulatory signals on distinct APC subsets (126, 127) and the chemical structure of iNKT cell agonists, which target iNKT cell ligands to distinct APC populations (128, 129) contribute to the functional outcome of iNKT cell activation. In serum, soluble iNKT cell agonists associate with lipoprotein particles or are transported bound to serum LTPs, which facilitate glycolipid uptake by APCs and loading onto CD1d molecules (130–132). Several receptors mediate the uptake of glycolipids for CD1d presentation, including the low-density lipoprotein receptor and the scavenger receptors SRA, SRB1, and CD36 (130, 132). Importantly, the specificity of this serum transport and receptor-mediated uptake is largely influenced by minor modifications of the chemical structure of iNKT cell agonists (133), suggesting that specific “targeting-motifs” could be used to direct glycolipid antigens toward distinct uptake pathways in order to modulate the resulting iNKT cell effector response (132). Besides affecting glycolipid uptake, the chemical structure of iNKT cell agonists may also influence the nature of the presenting APC as well as the context in which the antigen is CD1d presented. In general, the CD1d presentation of glycolipid antigens requires their access to the lysosomal loading compartment, which provides multiple glycosidases for antigen processing and lysosomal LTPs to assist in the solubilization and loading of glycolipids into CD1d (96–98, 134–136). There is evidence that lipid antigens eliciting a T_H_2-type iNKT cell cytokine response do not require intracellular loading onto CD1d but may directly bind surface CD1d instead. Such T_H_2-biased iNKT cell agonists typically possess short or unsaturated acyl chains, which increase their solubility in the aqueous environment but also favor a rapid displacement from CD1d upon recycling to the lysosome (137–140). The surface CD1d loading might bypass inclusion of such CD1d/lipid antigen complexes into lipid microdomains (139). Similarly, differential immune responses have been described for MHC class II molecules when presenting peptides either dependent or independent of lipid rafts (141). In addition, the anatomical context can modulate iNKT cell cytokine responses. In mice, the principle presenters of αGalCer and other T_H_1-biased antigens *in vivo* are CD8α^+^ DEC-205^+^ DCs (76), while the presentation of T_H_2-biased iNKT cell agonists was found to be more promiscuous, likely due to their ability to directly load onto cell surface CD1d (128). Furthermore, Lee et al. (142) showed that differential routes of lipid antigen application may dramatically alter the iNKT cell activation pattern due to a distinct anatomical distribution of iNKT cell subsets.

## Therapeutic Implications

**Figure 3:**
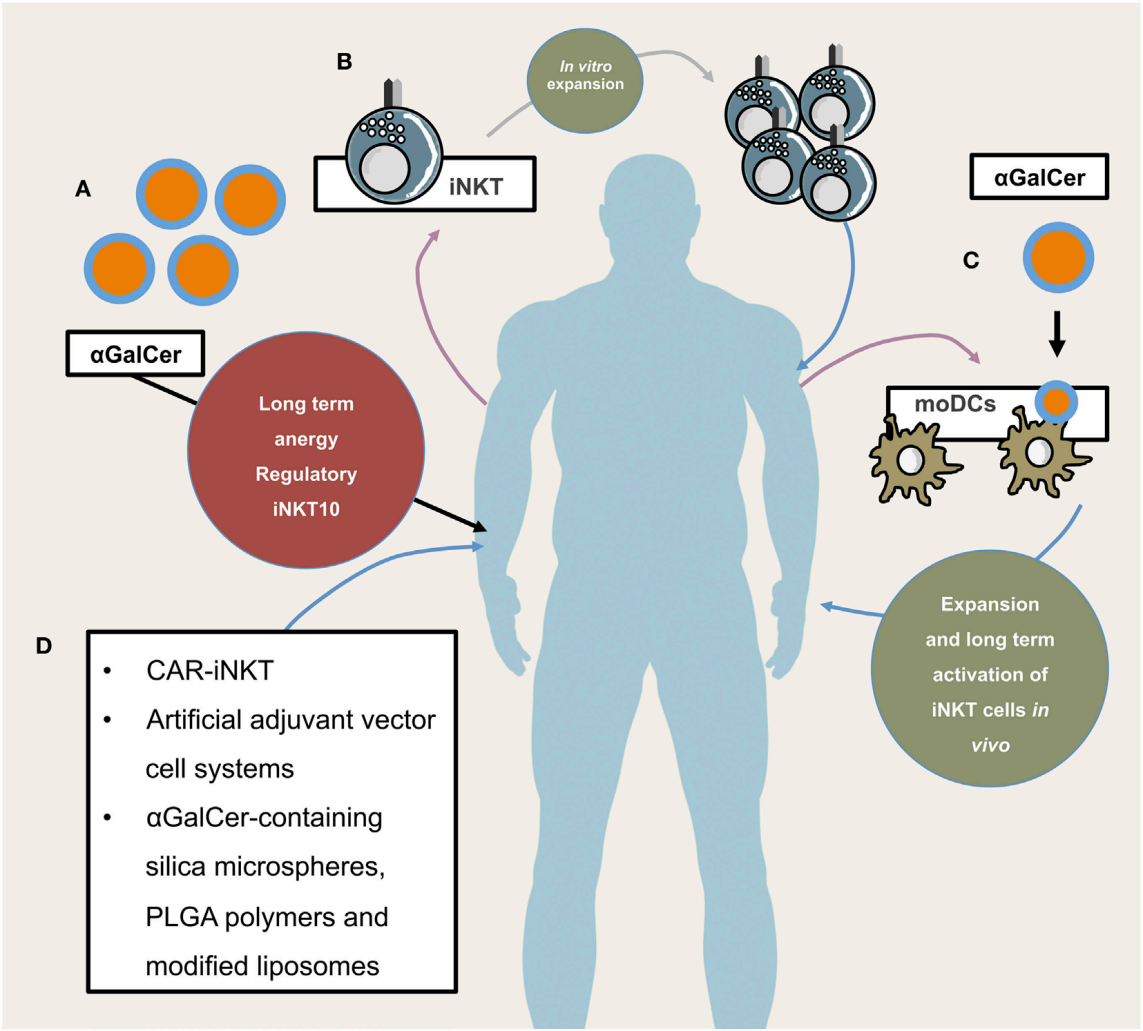
**Potential and already implemented invariant natural killer T cells (iNKT) cellular therapeutic approaches**. Illustrates different application modes of iNKT-based therapies. **(A)** Direct administration of iNKT cell agonist αGalCer. This has so far not proven to be efficacious due to long-term anergy and regulatory phenotype of iNKT cells following αGalCer administration. **(B)** Isolation, purification, *in vitro* expansion, and re-infusion of peripheral iNKT cells, **(C)** isolation of PBMCs and subsequent generation of monocyte-derived DCs which, after lipid-pulsing, are re-infused (either alone or in combination with *in vitro* expanded autologous iNKT cells), **(D)** novel and future approaches include chimeric antigen receptor (CAR)-iNKT cell generation, artificial adjuvant vector cell systems, αGalCer-containing silica microspheres, PLGA polymers, and modified liposomes.

Their ability to mature DCs and to transactivate both CTLs and NK cells for tumor cell eradication (143, 144) reflect the potential of iNKT cells in improving cancer immunotherapy (Figure 3). However, in contrast to encouraging studies performed in experimental models (145), clinical trials using direct administration of soluble αGalCer in cancer patients failed to show promising results (146). Aside from high interindividual variability in iNKT cell frequencies and inefficient targeting of particular subsets of lipid presenting cells, direct administration of antigenic glycolipids was shown to induce PD1:PDL1-dependent long-term anergy (147–149) or induction of regulatory IL-10-producing iNKT cells (36, 150), which negatively affect antitumor responses (150). As an alternative to αGalCer administration, DCs can be glycolipid-pulsed *ex vivo* followed by re-infusion. This strategy has proven to induce prolonged activation of iNKT cells rather than a regulatory/anergic phenotype, inhibits metastasis in an experimental melanoma model, and can expand human iNKT cells *in vivo* (78, 151, 152). Additionally, adoptive transfer of αGalCer-pulsed matured DCs expands iNKT cells in advanced stage cancer patients (153). A clinical phase I study in a limited number of individuals with metastatic malignancies reported that transfer of immature monocyte-derived DCs loaded with αGalCer was associated with a stronger recall response (154). Matured DCs as compared to immature DCs increased the observed beneficial effects significantly (153, 154). Another phase I trial during which patients with head and neck squamous cell carcinoma (HNSCC) were treated *via* singular co-administration of autologous *in vitro* expanded iNKT cells (intraarterial) and submucosal application of αGalCer-loaded APCs showed partial clinical response (155). In a small phase II clinical study in HNSCC patients using the same treatment regimen, 50% of the patients depicted tumor regression while 50% showed stable disease (156). Promising results were reported from a phase I-II study in non-small cell lung cancer patients: sequential intravenous administration of αGalCer-pulsed PBMCs increased the frequencies of IFNγ-producing cells in a majority of patients. This iNKT cell-mediated T_H_1 skewing in responders was associated with significantly prolonged median survival time (157). In a follow-up study, two candidate genes, *LTB4DH* and *DPYSL3*, were proposed to predict responsiveness to abovementioned treatment regimen (158). Late stage cancer patients often times are immune suppressed and retrieving enough APCs from these individuals for autologous transfer might prove difficult. Therefore, novel artificial APC constructs may help to circumvent lack of appropriate autologous APC numbers (85–87). Moreover, novel glycolipid-antigen delivery systems that systematically target relevant APC populations are currently being investigated. Some of these nanovector systems already show promising results. αGalCer-containing silica microspheres, poly(lactic-co-glycolic acid) (PLGA) polymers, and modified liposomes have already been reported to efficiently elicit iNKT cell responses (159–161). In order to initiate *in situ* responses of DCs, artificial adjuvant vector cell systems have been recently introduced. Herein, allogeneic CD1d-expressing NIH3T3 fibroblasts loaded with αGalCer were transfected with target-antigen mRNA. Injection of NIH3T3 fibroblasts lead to activation of iNKT cells, consecutive maturation of DCs, and activation of NK cells and antigen-specific CTLs. Animals that were immunized with adjuvant vector cells show potent immunity against antigen-bearing tumors. Interestingly, memory CTL responses can still be detected 12 months after initial single injection (143, 162).

Humans, as compared to mice, show high interindividual variability in iNKT cell frequencies. Patients with low steady-state numbers of iNKT cells might not efficiently profit from autologous transfer of lipid-pulsed APCs. Mouse embryonic fibroblast-derived induced pluripotent stem cells (iPSCs) can readily differentiate into functional iNKT cells. These iPSC-derived iNKT cells are able to produce IFNγ and mediate anti-neoplastic effects *in vivo* (163). Therefore, patients with low iNKT cell frequencies may be reconstituted with iPSC-derived iNKT cells as an efficient means to fully harness their immunomodulatory potential (143).

First attempts in using engineered iNKT cells with chimeric antigen receptors (CARs) show promising results. CAR-bearing iNKT cells home to designated tumor sites, eradicate tumor cells, and effectively execute cytotoxicity against TAMs without inducing graft-versus-host disease (164). Additionally, CD62L^+^ CD19-specific CAR-bearing iNKT cells show potent immunotherapeutic efficacy in a B cell lymphoma model (165).

In conclusion, murine and clinical trials performed to date demonstrate that therapeutic strategies that harness the biology of iNKT cells are generally well tolerated and, in some cases, effective in inducing tumor regression and prolonged survival. All of the tested and currently investigated strategies harness both the powerful cytolytic and adjuvant activity of iNKT cells in order to enhance protective antitumor immune responses. In order to fully exploit their therapeutic potential, it will not only be essential to elucidate the differential effector functions and modes of activation of individual iNKT cell subsets but also the immunological contexts and transcriptional programs that direct CD24^hi^/CD69^+^ iNKT cell progenitors into development toward specific iNKT cell subpopulations as well as determinants that gear specific iNKT cell subsets to distinct anatomical sites (31, 142, 166). Profound mechanistic insight into understanding how DCs activate and instruct iNKT cells and which factors regulate iNKT cell responses are prerequisites for improving the efficacy of iNKT cell-targeting therapies. In addition, clinical trials will be instrumental in identifying the optimal ligands and APC populations to induce vigorous iNKT cell activation and in determining the routes and intervals of administration to achieve sustained antitumor immunity.

## Author Contributions

CWK, SF, and JDL participated in drafting the article and revising it critically for important intellectual content. All authors gave final approval of the submitted manuscript.

## Conflict of Interest Statement

The authors declare that the research was conducted in the absence of any commercial or financial relationships that could be construed as a potential conflict of interest.

## References

[1] Godfrey DI Uldrich AP McCluskey J Rossjohn J Moody DB. The burgeoning family of unconventional T cells. Nat Immunol (2015) 16:1114–23. 10.1038/ni.3298 26482978

[2] Salio M Silk JD Jones EY Cerundolo V. Biology of CD1- and MR1-restricted T cells. Annu Rev Immunol (2014) 32:323–66. 10.1146/annurev-immunol-032713-120243 24499274

[3] Macho-Fernandez E Brigl M. The extended family of CD1d-restricted NKT cells: sifting through a mixed bag of TCRs, antigens, and functions. Front Immunol (2015) 6:362. 10.3389/fimmu.2015.00362 26284062 PMC4517383

[4] Marrero I Ware R Kumar V. Type II NKT cells in inflammation, autoimmunity, microbial immunity, and cancer. Front Immunol (2015) 6:316. 10.3389/fimmu.2015.00316 26136748 PMC4470258

[5] Budd RC Miescher GC Howe RC Lees RK Bron C MacDonald HR. Developmentally regulated expression of T cell receptor beta chain variable domains in immature thymocytes. J Exp Med (1987) 166:577–82. 10.1084/jem.166.2.577 3496420 PMC2189605

[6] Fowlkes BJ Kruisbeek AM Ton-That H Weston MA Coligan JE Schwartz RH A novel population of T-cell receptor alpha beta-bearing thymocytes which predominantly expresses a single V beta gene family. Nature (1987) 329:251–4. 10.1038/329251a0 3114646

[7] Imai K Kanno M Kimoto H Shigemoto K Yamamoto S Taniguchi M. Sequence and expression of transcripts of the T-cell antigen receptor alpha-chain gene in a functional, antigen-specific suppressor-T-cell hybridoma. Proc Natl Acad Sci U S A (1986) 83:8708–12. 10.1073/pnas.83.22.8708 2946043 PMC387000

[8] Kawano T Cui J Koezuka Y Toura I Kaneko Y Motoki K CD1d-restricted and TCR-mediated activation of valpha14 NKT cells by glycosylceramides. Science (1997) 278:1626–9. 10.1126/science.278.5343.1626 9374463

[9] Brennan PJ Brigl M Brenner MB. Invariant natural killer T cells: an innate activation scheme linked to diverse effector functions. Nat Rev Immunol (2013) 13:101–17. 10.1038/nri3369 23334244

[10] Taniguchi M Harada M Dashtsoodol N Kojo S. Discovery of NKT cells and development of NKT cell-targeted anti-tumor immunotherapy. Proc Jpn Acad Ser B Phys Biol Sci (2015) 91:292–304. 10.2183/pjab.91.292 26194854 PMC4631895

[11] Kuylenstierna C Björkström NK Andersson SK Sahlström P Bosnjak L Paquin-Proulx D NKG2D performs two functions in invariant NKT cells: direct TCR-independent activation of NK-like cytolysis and co-stimulation of activation by CD1d. Eur J Immunol (2011) 41:1913–23. 10.1002/eji.200940278 21590763 PMC3523190

[12] Shimizu K Sato Y Shinga J Watanabe T Endo T Asakura M KLRG+ invariant natural killer T cells are long-lived effectors. Proc Natl Acad Sci U S A (2014) 111:12474–9. 10.1073/pnas.1406240111 25118276 PMC4151776

[13] Brigl M Tatituri RV Watts GF Bhowruth V Leadbetter EA Barton N Innate and cytokine-driven signals, rather than microbial antigens, dominate in natural killer T cell activation during microbial infection. J Exp Med (2011) 208:1163–77. 10.1084/jem.20102555 21555485 PMC3173255

[14] Benlagha K Weiss A Beavis A Teyton L Bendelac A. In vivo identification of glycolipid antigen-specific T cells using fluorescent CD1d tetramers. J Exp Med (2000) 191:1895–903. 10.1084/jem.191.11.1895 10839805 PMC2213523

[15] Matsuda JL Naidenko OV Gapin L Nakayama T Taniguchi M Wang CR Tracking the response of natural killer T cells to a glycolipid antigen using CD1d tetramers. J Exp Med (2000) 192:741–54. 10.1084/jem.192.5.741 10974039 PMC2193268

[16] Godfrey DI Macdonald HR Kronenberg M Smyth MJ Van Kaer L NKT cells: what’s in a name? Nat Rev Immunol (2004) 4:231–7. 10.1038/nri1309 15039760

[17] Bendelac A Lantz O Quimby ME Yewdell JW Bennink JR Brutkiewicz RR. CD1 recognition by mouse NK1+ T lymphocytes. Science (1995) 268:863–5. 10.1126/science.7538697 7538697

[18] Bendelac A Savage PB Teyton L. The biology of NKT cells. Annu Rev Immunol (2007) 25:297–336. 10.1146/annurev.immunol.25.022106.141711 17150027

[19] Carnaud C Lee D Donnars O Park SH Beavis A Koezuka Y Cutting edge: cross-talk between cells of the innate immune system: NKT cells rapidly activate NK cells. J Immunol (1999) 163:4647–50. 10528160

[20] Chang P-P Barral P Fitch J Pratama A Ma CS Kallies A Identification of Bcl-6-dependent follicular helper NKT cells that provide cognate help for B cell responses. Nat Immunol (2012) 13:35–43. 10.1038/ni.2166 22120117

[21] Brossay L Chioda M Burdin N Koezuka Y Casorati G Dellabona P CD1d-mediated recognition of an alpha-galactosylceramide by natural killer T cells is highly conserved through mammalian evolution. J Exp Med (1998) 188:1521–8. 10.1084/jem.188.8.1521 9782129 PMC2213408

[22] Hammond KJ Kronenberg M. Natural killer T cells: natural or unnatural regulators of autoimmunity? Curr Opin Immunol (2003) 15:683–9. 10.1016/j.coi.2003.09.014 14630203

[23] Schipper HS Rakhshandehroo M van de Graaf SF Venken K Koppen A Stienstra R Natural killer T cells in adipose tissue prevent insulin resistance. J Clin Invest (2012) 122:3343–54. 10.1172/JCI62739 22863618 PMC3428087

[24] Watarai H Nakagawa R Omori-Miyake M Dashtsoodol N Taniguchi M. Methods for detection, isolation and culture of mouse and human invariant NKT cells. Nat Protoc (2008) 3:70–8. 10.1038/nprot.2007.515 18193023

[25] Berzins SP Smyth MJ Baxter AG. Presumed guilty: natural killer T cell defects and human disease. Nat Rev Immunol (2011) 11:131–42. 10.1038/nri2904 21267014

[26] Moran AE Holzapfel KL Xing Y Cunningham NR Maltzman JS Punt J T cell receptor signal strength in Treg and iNKT cell development demonstrated by a novel fluorescent reporter mouse. J Exp Med (2011) 208:1279–89. 10.1084/jem.20110308 21606508 PMC3173240

[27] Lee YJ Holzapfel KL Zhu J Jameson SC Hogquist KA. Steady-state production of IL-4 modulates immunity in mouse strains and is determined by lineage diversity of iNKT cells. Nat Immunol (2013) 14:1146–54. 10.1038/ni.2731 24097110 PMC3824254

[28] Chiu YH Park SH Benlagha K Forestier C Jayawardena-Wolf J Savage PB Multiple defects in antigen presentation and T cell development by mice expressing cytoplasmic tail-truncated CD1d. Nat Immunol (2002) 3:55–60. 10.1038/ni740 11731798

[29] Sandberg JK Stoddart CA Brilot F Jordan KA Nixon DF. Development of innate CD4+ alpha-chain variable gene segment 24 (Valpha24) natural killer T cells in the early human fetal thymus is regulated by IL-7. Proc Natl Acad Sci U S A (2004) 101:7058–63. 10.1073/pnas.0305986101 15118099 PMC406465

[30] Seiler MP Mathew R Liszewski MK Spooner CJ Barr K Meng F Elevated and sustained expression of the transcription factors Egr1 and Egr2 controls NKT lineage differentiation in response to TCR signaling. Nat Immunol (2012) 13:264–71. 10.1038/ni.2230 22306690 PMC3288314

[31] Gapin L. Development of invariant natural killer T cells. Curr Opin Immunol (2016) 39:68–74. 10.1016/j.coi.2016.01.001 26802287 PMC4801673

[32] Kim EY Lynch L Brennan PJ Cohen NR Brenner MB. The transcriptional programs of iNKT cells. Semin Immunol (2015) 27:26–32. 10.1016/j.smim.2015.02.005 25841627 PMC6322908

[33] Savage AK Constantinides MG Han J Picard D Martin E Li B The transcription factor PLZF directs the effector program of the NKT cell lineage. Immunity (2008) 29:391–403. 10.1016/j.immuni.2008.07.011 18703361 PMC2613001

[34] Kovalovsky D Uche OU Eladad S Hobbs RM Yi W Alonzo E The BTB-zinc finger transcriptional regulator PLZF controls the development of invariant natural killer T cell effector functions. Nat Immunol (2008) 9:1055–64. 10.1038/ni.1641 18660811 PMC2662733

[35] Engel I Seumois G Chavez L Samaniego-Castruita D White B Chawla A Innate-like functions of natural killer T cell subsets result from highly divergent gene programs. Nat Immunol (2016) 17:728–39. 10.1038/ni.3437 27089380 PMC4944658

[36] Lynch L Michelet X Zhang S Brennan PJ Moseman A Lester C Regulatory iNKT cells lack expression of the transcription factor PLZF and control the homeostasis of T(reg) cells and macrophages in adipose tissue. Nat Immunol (2015) 16:85–95. 10.1038/ni.3047 25436972 PMC4343194

[37] Lynch L Nowak M Varghese B Clark J Hogan AE Toxavidis V Adipose tissue invariant NKT cells protect against diet-induced obesity and metabolic disorder through regulatory cytokine production. Immunity (2012) 37:574–87. 10.1016/j.immuni.2012.06.016 22981538 PMC4991771

[38] Kanda M Yamanaka H Kojo S Usui Y Honda H Sotomaru Y Transcriptional regulator Bhlhe40 works as a cofactor of T-bet in the regulation of IFN-γ production in iNKT cells. Proc Natl Acad Sci U S A (2016) 113(24):E3394–402. 10.1073/pnas.1604178113 27226296 PMC4914147

[39] Brigl M Bry L Kent SC Gumperz JE Brenner MB. Mechanism of CD1d-restricted natural killer T cell activation during microbial infection. Nat Immunol (2003) 4:1230–7. 10.1038/ni1002 14578883

[40] Mattner J Debord KL Ismail N Goff RD Cantu C Zhou D Exogenous and endogenous glycolipid antigens activate NKT cells during microbial infections. Nature (2005) 434:525–9. 10.1038/nature03408 15791258

[41] Wilson MT Johansson C Olivares-Villagómez D Singh AK Stanic AK Wang C-R The response of natural killer T cells to glycolipid antigens is characterized by surface receptor down-modulation and expansion. Proc Natl Acad Sci U S A (2003) 100:10913–8. 10.1073/pnas.1833166100 12960397 PMC196902

[42] Smyth MJ Wallace ME Nutt SL Yagita H Godfrey DI Hayakawa Y. Sequential activation of NKT cells and NK cells provides effective innate immunotherapy of cancer. J Exp Med (2005) 201:1973–85. 10.1084/jem.20042280 15967825 PMC1364507

[43] Smyth MJ Crowe NY Pellicci DG Kyparissoudis K Kelly JM Takeda K Sequential production of interferon-gamma by NK1.1(+) T cells and natural killer cells is essential for the antimetastatic effect of alpha-galactosylceramide. Blood (2002) 99:1259–66. 10.1182/blood.V99.4.1259 11830474

[44] Schneiders FL de Bruin RC Santegoets SJ Bonneville M Scotet E Scheper RJ Activated iNKT cells promote Vγ9Vδ2-T cell anti-tumor effector functions through the production of TNF-α. Clin Immunol (2012) 142:194–200. 10.1016/j.clim.2011.10.006 22122798

[45] Paget C Bialecki E Fontaine J Vendeville C Mallevaey T Faveeuw C Role of invariant NK T lymphocytes in immune responses to CpG oligodeoxynucleotides. J Immunol (2009) 182:1846–53. 10.4049/jimmunol.0802492 19201836

[46] Leadbetter EA Brigl M Illarionov P Cohen N Luteran MC Pillai S NK T cells provide lipid antigen-specific cognate help for B cells. Proc Natl Acad Sci U S A (2008) 105:8339–44. 10.1073/pnas.0801375105 18550809 PMC2448838

[47] King IL Fortier A Tighe M Dibble J Watts GFM Veerapen N Invariant natural killer T cells direct B cell responses to cognate lipid antigen in an IL-21-dependent manner. Nat Immunol (2012) 13:44–50. 10.1038/ni.2172 PMC3833037 22120118

[48] Bialecki E Paget C Fontaine J Capron M Trottein F Faveeuw C. Role of marginal zone B lymphocytes in invariant NKT cell activation. J Immunol (2009) 182:6105–13. 10.4049/jimmunol.0802273 19414762

[49] Zietara N Łyszkiewicz M Krueger A Weiss S. ICOS-dependent stimulation of NKT cells by marginal zone B cells. Eur J Immunol (2011) 41:3125–34. 10.1002/eji.201041092 21809338

[50] Vomhof-Dekrey EE Yates J Hägglöf T Lanthier P Amiel E Veerapen N Cognate interaction with iNKT cells expands IL-10-producing B regulatory cells. Proc Natl Acad Sci U S A (2015) 112:12474–9. 10.1073/pnas.1504790112 26392556 PMC4603516

[51] Crowe NY Uldrich AP Kyparissoudis K Hammond KJ Hayakawa Y Sidobre S Glycolipid antigen drives rapid expansion and sustained cytokine production by NK T cells. J Immunol (2003) 171:4020–7. 10.4049/jimmunol.171.8.4020 14530322

[52] Stetson DB Mohrs M Reinhardt RL Baron JL Wang ZE Gapin L Constitutive cytokine mRNAs mark natural killer (NK) and NK T cells poised for rapid effector function. J Exp Med (2003) 198:1069–76. 10.1084/jem.20030630 14530376 PMC2194220

[53] Matsuda JL Gapin L Baron JL Sidobre S Stetson DB Mohrs M Mouse V alpha 14i natural killer T cells are resistant to cytokine polarization in vivo. Proc Natl Acad Sci U S A (2003) 100:8395–400. 10.1073/pnas.1332805100 12829795 PMC166240

[54] Nagaleekar VK Sabio G Aktan I Chant A Howe IW Thornton TM Translational control of NKT cell cytokine production by p38 MAPK. J Immunol (2011) 186:4140–6. 10.4049/jimmunol.1002614 21368234 PMC3697841

[55] Wingender G Krebs P Beutler B Kronenberg M. Antigen-specific cytotoxicity by invariant NKT cells in vivo is CD95/CD178-dependent and is correlated with antigenic potency. J Immunol (2010) 185:2721–9. 10.4049/jimmunol.1001018 20660713 PMC2989418

[56] Kumar V Patel S Tcyganov E Gabrilovich DI. The nature of myeloid-derived suppressor cells in the tumor microenvironment. Trends Immunol (2016) 37:208–20. 10.1016/j.it.2016.01.004 26858199 PMC4775398

[57] De Santo C Salio M Masri SH Lee LY Dong T Speak AO Invariant NKT cells reduce the immunosuppressive activity of influenza A virus-induced myeloid-derived suppressor cells in mice and humans. J Clin Invest (2008) 118:4036–48. 10.1172/JCI36264 19033672 PMC2582442

[58] Ko HJ Lee JM Kim YJ Kim YS Lee KA Kang CY. Immunosuppressive myeloid-derived suppressor cells can be converted into immunogenic APCs with the help of activated NKT cells: an alternative cell-based antitumor vaccine. J Immunol (2009) 182:1818–28. 10.4049/jimmunol.0802430 19201833

[59] Gabrilovich DI Bronte V Chen SH Colombo MP Ochoa A Ostrand-Rosenberg S The terminology issue for myeloid-derived suppressor cells. Cancer Res (2007) 67:425; author reply 426 10.1158/0008-5472.CAN-06-3037 17210725 PMC1941787

[60] De Santo C Arscott R Booth S Karydis I Jones M Asher R Invariant NKT cells modulate the suppressive activity of IL-10-secreting neutrophils differentiated with serum amyloid A. Nat Immunol (2010) 11:1039–46. 10.1038/ni.1942 20890286 PMC3001335

[61] Kusmartsev S Su Z Heiser A Dannull J Eruslanov E Kübler H Reversal of myeloid cell-mediated immunosuppression in patients with metastatic renal cell carcinoma. Clin Cancer Res (2008) 14:8270–8. 10.1158/1078-0432.CCR-08-0165 19088044

[62] Kusmartsev S Cheng F Yu B Nefedova Y Sotomayor E Lush R All-trans-retinoic acid eliminates immature myeloid cells from tumor-bearing mice and improves the effect of vaccination. Cancer Res (2003) 63:4441–9. 12907617

[63] Lee JM Seo JH Kim YJ Kim YS Ko HJ Kang CY. The restoration of myeloid-derived suppressor cells as functional antigen-presenting cells by NKT cell help and all-trans-retinoic acid treatment. Int J Cancer (2012) 131:741–51. 10.1002/ijc.26411 21898392

[64] Gabrilovich DI Nagaraj S. Myeloid-derived suppressor cells as regulators of the immune system. Nat Rev Immunol (2009) 9:162–74. 10.1038/nri2506 19197294 PMC2828349

[65] Song L Asgharzadeh S Salo J Engell K Wu HW Sposto R Valpha24-invariant NKT cells mediate antitumor activity via killing of tumor-associated macrophages. J Clin Invest (2009) 119:1524–36. 10.1172/JCI37869 19411762 PMC2689106

[66] Nieda M Nicol A Koezuka Y Kikuchi A Lapteva N Tanaka Y TRAIL expression by activated human CD4(+)V alpha 24NKT cells induces in vitro and in vivo apoptosis of human acute myeloid leukemia cells. Blood (2001) 97:2067–74. 10.1182/blood.V97.7.2067 11264173

[67] Lauwerys BR Garot N Renauld JC Houssiau FA. Cytokine production and killer activity of NK/T-NK cells derived with IL-2, IL-15, or the combination of IL-12 and IL-18. J Immunol (2000) 165:1847–53. 10.4049/jimmunol.165.4.1847 10925263

[68] Metelitsa LS Naidenko OV Kant A Wu HW Loza MJ Perussia B Human NKT cells mediate antitumor cytotoxicity directly by recognizing target cell CD1d with bound ligand or indirectly by producing IL-2 to activate NK cells. J Immunol (2001) 167:3114–22. 10.4049/jimmunol.167.6.3114 11544296

[69] Mori L Lepore M De Libero G. The immunology of CD1- and MR1-restricted T cells. Annu Rev Immunol (2016) 34:479–510. 10.1146/annurev-immunol-032414-112008 26927205

[70] Sköld M Xiong X Illarionov PA Besra GS Behar SM. Interplay of cytokines and microbial signals in regulation of CD1d expression and NKT cell activation. J Immunol (2005) 175:3584–93. 10.4049/jimmunol.175.6.3584 16148102

[71] Busshoff U Hein A Iglesias A Dörries R Régnier-Vigouroux A. CD1 expression is differentially regulated by microglia, macrophages and T cells in the central nervous system upon inflammation and demyelination. J Neuroimmunol (2001) 113:220–30. 10.1016/S0165-5728(00)00450-1 11164905

[72] de Lalla C Galli G Aldrighetti L Romeo R Mariani M Monno A Production of profibrotic cytokines by invariant NKT cells characterizes cirrhosis progression in chronic viral hepatitis. J Immunol (2004) 173:1417–25. 10.4049/jimmunol.173.2.1417 15240738

[73] Canchis PW Bhan AK Landau SB Yang L Balk SP Blumberg RS Tissue distribution of the non-polymorphic major histocompatibility complex class I-like molecule, CD1d. Immunology (1993) 80:561–5. 7508419 PMC1422252

[74] Balk SP Burke S Polischuk JE Frantz ME Yang L Porcelli S Beta 2-microglobulin-independent MHC class Ib molecule expressed by human intestinal epithelium. Science (1994) 265:259–62. 10.1126/science.7517575 7517575

[75] Blumberg RS Terhorst C Bleicher P McDermott FV Allan CH Landau SB Expression of a nonpolymorphic MHC class I-like molecule, CD1D, by human intestinal epithelial cells. J Immunol (1991) 147:2518–24. 1717564

[76] Arora P Baena A Yu KO Saini NK Kharkwal SS Goldberg MF A single subset of dendritic cells controls the cytokine bias of natural killer T cell responses to diverse glycolipid antigens. Immunity (2014) 40:105–16. 10.1016/j.immuni.2013.12.004 24412610 PMC3895174

[77] Fujii S Liu K Smith C Bonito AJ Steinman RM. The linkage of innate to adaptive immunity via maturing dendritic cells in vivo requires CD40 ligation in addition to antigen presentation and CD80/86 costimulation. J Exp Med (2004) 199:1607–18. 10.1084/jem.20040317 15197224 PMC2212806

[78] Fujii S Shimizu K Kronenberg M Steinman RM. Prolonged IFN-gamma-producing NKT response induced with alpha-galactosylceramide-loaded DCs. Nat Immunol (2002) 3:867–74. 10.1038/ni827 12154358

[79] Paget C Mallevaey T Speak AO Torres D Fontaine J Sheehan KC Activation of invariant NKT cells by toll-like receptor 9-stimulated dendritic cells requires type I interferon and charged glycosphingolipids. Immunity (2007) 27:597–609. 10.1016/j.immuni.2007.08.017 17950005

[80] Darmoise A Teneberg S Bouzonville L Brady RO Beck M Kaufmann SH Lysosomal alpha-galactosidase controls the generation of self lipid antigens for natural killer T cells. Immunity (2010) 33:216–28. 10.1016/j.immuni.2010.08.003 20727792 PMC4018304

[81] Kain L Webb B Anderson BL Deng S Holt M Constanzo A The identification of the endogenous ligands of natural killer T cells reveals the presence of mammalian &alpha;-linked glycosylceramides. Immunity (2014) 41:543–54. 10.1016/j.immuni.2014.08.017 25367571 PMC4220304

[82] Holzapfel KL Tyznik AJ Kronenberg M Hogquist KA. Antigen-dependent versus -independent activation of invariant NKT cells during infection. J Immunol (2014) 192:5490–8. 10.4049/jimmunol.1400722 24813205 PMC4053538

[83] Kitamura H Iwakabe K Yahata T Nishimura S Ohta A Ohmi Y The natural killer T (NKT) cell ligand alpha-galactosylceramide demonstrates its immunopotentiating effect by inducing interleukin (IL)-12 production by dendritic cells and IL-12 receptor expression on NKT cells. J Exp Med (1999) 189:1121–8. 10.1084/jem.189.7.1121 10190903 PMC2193012

[84] Hayakawa Y Takeda K Yagita H Van Kaer L Saiki I Okumura K. Differential regulation of Th1 and Th2 functions of NKT cells by CD28 and CD40 costimulatory pathways. J Immunol (2001) 166:6012–8. 10.4049/jimmunol.166.10.6012 11342617

[85] Sun W Subrahmanyam PB East JE Webb TJ. Connecting the dots: artificial antigen presenting cell-mediated modulation of natural killer T cells. J Interferon Cytokine Res (2012) 32:505–16. 10.1089/jir.2012.0045 23050947 PMC3493043

[86] Shiratsuchi T Schneck J Kawamura A Tsuji M. Human CD1 dimeric proteins as indispensable tools for research on CD1-binding lipids and CD1-restricted T cells. J Immunol Methods (2009) 345:49–59. 10.1016/j.jim.2009.04.002 19374905 PMC2743114

[87] East JE Sun W Webb TJ. Artificial antigen presenting cell (aAPC) mediated activation and expansion of natural killer T cells. J Vis Exp (2012) (70):e4333. 10.3791/4333 23299308 PMC3577866

[88] Kim HS Garcia J Exley M Johnson KW Balk SP Blumberg RS. Biochemical characterization of CD1d expression in the absence of beta2-microglobulin. J Biol Chem (1999) 274:9289–95. 10.1074/jbc.274.14.9289 10092605

[89] Dougan SK Salas A Rava P Agyemang A Kaser A Morrison J Microsomal triglyceride transfer protein lipidation and control of CD1d on antigen-presenting cells. J Exp Med (2005) 202:529–39. 10.1084/jem.20050183 16087713 PMC2212858

[90] Kang SJ Cresswell P. Calnexin, calreticulin, and ERp57 cooperate in disulfide bond formation in human CD1d heavy chain. J Biol Chem (2002) 277:44838–44. 10.1074/jbc.M207831200 12239218

[91] Elewaut D Lawton AP Nagarajan NA Maverakis E Khurana A Honing S The adaptor protein AP-3 is required for CD1d-mediated antigen presentation of glycosphingolipids and development of V 14i NKT cells. J Exp Med (2003) 198:1133–46. 10.1084/jem.20030143 14557411 PMC2194227

[92] Cernadas M Sugita M van der Wel N Cao X Gumperz JE Maltsev S Lysosomal localization of murine CD1d mediated by AP-3 is necessary for NK T cell development. J Immunol (2003) 171:4149–55. 10.4049/jimmunol.171.8.4149 14530337

[93] Vartabedian VF Savage PB Teyton L. The processing and presentation of lipids and glycolipids to the immune system. Immunol Rev (2016) 272:109–19. 10.1111/imr.12431 27319346 PMC4916853

[94] Keller CW Loi M Ewert S Quast I Theiler R Gannagé M The autophagy machinery restrains iNKT cell activation through CD1D1 internalization. Autophagy (2017) 15:1–12. 10.1080/15548627.2017.1297907 28296542 PMC5486365

[95] Sille FCM Boxem M Sprengers D Veerapen N Besra G Boes M Distinct requirements for CD1d intracellular transport for development of V 14 iNKT cells. J Immunol (2009) 183:1780–8. 10.4049/jimmunol.0901354 19587020 PMC2839504

[96] Zhou D Cantu C Sagiv Y Schrantz N Kulkarni AB Qi X Editing of CD1d-bound lipid antigens by endosomal lipid transfer proteins. Science (2004) 303:523–7. 10.1126/science.1092009 14684827 PMC2918537

[97] Kang SJ Cresswell P. Saposins facilitate CD1d-restricted presentation of an exogenous lipid antigen to T cells. Nat Immunol (2004) 5:175–81. 10.1038/ni1034 14716312

[98] Schrantz N Sagiv Y Liu Y Savage PB Bendelac A Teyton L. The Niemann-Pick type C2 protein loads isoglobotrihexosylceramide onto CD1d molecules and contributes to the thymic selection of NKT cells. J Exp Med (2007) 204:841–52. 10.1084/jem.20061562 17389239 PMC2118543

[99] Sagiv Y Hudspeth K Mattner J Schrantz N Stern RK Zhou D Cutting edge: impaired glycosphingolipid trafficking and NKT cell development in mice lacking Niemann-Pick type C1 protein. J Immunol (2006) 177:26–30. 10.4049/jimmunol.177.1.26 16785493

[100] Ly D Moody DB. The CD1 size problem: lipid antigens, ligands, and scaffolds. Cell Mol Life Sci (2014) 71:3069–79. 10.1007/s00018-014-1603-6 24658584 PMC4160407

[101] Zeissig S Murata K Sweet L Publicover J Hu Z Kaser A Hepatitis B virus-induced lipid alterations contribute to natural killer T cell-dependent protective immunity. Nat Med (2012) 18:1060–8. 10.1038/nm.2811 22706385 PMC3478098

[102] Stern LJ Santambrogio L. The melting pot of the MHC II peptidome. Curr Opin Immunol (2016) 40:70–7. 10.1016/j.coi.2016.03.004 27018930 PMC4884503

[103] Cox D Fox L Tian R Bardet W Skaley M Mojsilovic D Determination of cellular lipids bound to human CD1d molecules. PLoS One (2009) 4:e5325. 10.1371/journal.pone.0005325 19415116 PMC2673035

[104] Chiu YH Jayawardena J Weiss A Lee D Park SH Dautry-Varsat A Distinct subsets of CD1d-restricted T cells recognize self-antigens loaded in different cellular compartments. J Exp Med (1999) 189:103–10. 10.1084/jem.189.1.103 9874567 PMC1887692

[105] Zhou D Mattner J Cantu C Schrantz N Yin N Gao Y Lysosomal glycosphingolipid recognition by NKT cells. Science (2004) 306:1786–9. 10.1126/science.1103440 15539565

[106] Speak AO Salio M Neville DC Fontaine J Priestman DA Platt N Implications for invariant natural killer T cell ligands due to the restricted presence of isoglobotrihexosylceramide in mammals. Proc Natl Acad Sci U S A (2007) 104:5971–6. 10.1073/pnas.0607285104 17372214 PMC1851601

[107] Porubsky S Speak AO Luckow B Cerundolo V Platt FM Gröne H-J. Normal development and function of invariant natural killer T cells in mice with isoglobotrihexosylceramide (iGb3) deficiency. Proc Natl Acad Sci U S A (2007) 104:5977–82. 10.1073/pnas.0611139104 17372206 PMC1851602

[108] Facciotti F Ramanjaneyulu GS Lepore M Sansano S Cavallari M Kistowska M Peroxisome-derived lipids are self antigens that stimulate invariant natural killer T cells in the thymus. Nat Immunol (2012) 13:474–80. 10.1038/ni.2245 22426352

[109] Kinjo Y Tupin E Wu D Fujio M Garcia-Navarro R Benhnia MR Natural killer T cells recognize diacylglycerol antigens from pathogenic bacteria. Nat Immunol (2006) 7:978–86. 10.1038/ni1380 16921381

[110] Kinjo Y Wu D Kim G Xing G-W Poles MA Ho DD Recognition of bacterial glycosphingolipids by natural killer T cells. Nature (2005) 434:520–5. 10.1038/nature03407 15791257

[111] Sriram V Du W Gervay-Hague J Brutkiewicz RR. Cell wall glycosphingolipids of *Sphingomonas paucimobilis* are CD1d-specific ligands for NKT cells. Eur J Immunol (2005) 35:1692–701. 10.1002/eji.200526157 15915536

[112] Kinjo Y Illarionov P Véla JL Pei B Girardi E Li X Invariant natural killer T cells recognize glycolipids from pathogenic Gram-positive bacteria. Nat Immunol (2011) 12:966–74. 10.1038/ni.2096 21892173 PMC3178673

[113] Fischer K Scotet E Niemeyer M Koebernick H Zerrahn J Maillet S Mycobacterial phosphatidylinositol mannoside is a natural antigen for CD1d-restricted T cells. Proc Natl Acad Sci U S A (2004) 101:10685–90. 10.1073/pnas.0403787101 15243159 PMC489995

[114] Ito Y Véla JL Matsumura F Hoshino H Tyznik A Lee H *Helicobacter pylori* cholesteryl α-glucosides contribute to its pathogenicity and immune response by natural killer T cells. PLoS One (2013) 8:e78191. 10.1371/journal.pone.0078191 24312443 PMC3846475

[115] Wieland Brown LC Penaranda C Kashyap PC Williams BB Clardy J Kronenberg M Production of α-galactosylceramide by a prominent member of the human gut microbiota. PLoS Biol (2013) 11:e1001610. 10.1371/journal.pbio.1001610 23874157 PMC3712910

[116] Natori T Koezuka Y Higa T Agelasphins, novel α-galactosylceramides from the marine sponge *Agelas* mauritianus. Tetrahedron Lett (1993) 34:5591–2. 10.1016/S0040-4039(00)73889-5

[117] Albacker LA Chaudhary V Chang Y-J Kim HY Chuang YT Pichavant M Invariant natural killer T cells recognize a fungal glycosphingolipid that can induce airway hyperreactivity. Nat Med (2013) 19(10):1297–304. 10.1038/nm.3321 23995283 PMC4079117

[118] Fujii S Shimizu K Smith C Bonifaz L Steinman RM. Activation of natural killer T cells by alpha-galactosylceramide rapidly induces the full maturation of dendritic cells in vivo and thereby acts as an adjuvant for combined CD4 and CD8 T cell immunity to a coadministered protein. J Exp Med (2003) 198:267–79. 10.1084/jem.20030324 12874260 PMC2194082

[119] Hermans IF Silk JD Gileadi U Salio M Mathew B Ritter G NKT cells enhance CD4+ and CD8+ T cell responses to soluble antigen in vivo through direct interaction with dendritic cells. J Immunol (2003) 171:5140–7. 10.4049/jimmunol.171.10.5140 14607913

[120] Haan den JM Lehar SM Bevan MJ. CD8(+) but not CD8(-) dendritic cells cross-prime cytotoxic T cells in vivo. J Exp Med (2000) 192:1685–96. 10.1084/jem.192.12.1685 11120766 PMC2213493

[121] Bennett SR Carbone FR Karamalis F Miller JF Heath WR. Induction of a CD8+ cytotoxic T lymphocyte response by cross-priming requires cognate CD4+ T cell help. J Exp Med (1997) 186:65–70. 10.1084/jem.186.1.65 9206998 PMC2198964

[122] Aarntzen EH de Vries IJ Lesterhuis WJ Schuurhuis D Jacobs JF Bol K Targeting CD4(+) T-helper cells improves the induction of antitumor responses in dendritic cell-based vaccination. Cancer Res (2013) 73:19–29. 10.1158/0008-5472.CAN-12-1127 23087058

[123] Castellino F Huang AY Altan-Bonnet G Stoll S Scheinecker C Germain RN. Chemokines enhance immunity by guiding naive CD8+ T cells to sites of CD4+ T cell-dendritic cell interaction. Nature (2006) 440:890–5. 10.1038/nature04651 16612374

[124] Semmling V Lukacs-Kornek V Thaiss CA Quast T Hochheiser K Panzer U Alternative cross-priming through CCL17-CCR4-mediated attraction of CTLs toward NKT cell-licensed DCs. Nat Immunol (2010) 11:313–20. 10.1038/ni.1848 20190758

[125] Arora P Venkataswamy MM Baena A Bricard G Li Q Veerapen N A rapid fluorescence-based assay for classification of iNKT cell activating glycolipids. J Am Chem Soc (2011) 133:5198–201. 10.1021/ja200070u 21425779 PMC3072113

[126] Im JS Tapinos N Chae G-T Illarionov PA Besra GS DeVries GH Expression of CD1d molecules by human schwann cells and potential interactions with immunoregulatory invariant NK T cells. J Immunol (2006) 177:5226–35. 10.4049/jimmunol.177.8.5226 17015708

[127] Bezbradica JS Stanic AK Matsuki N Bour-Jordan H Bluestone JA Thomas JW Distinct roles of dendritic cells and B cells in Va14Ja18 natural T cell activation in vivo. J Immunol (2005) 174:4696–705. 10.4049/jimmunol.174.8.4696 15814694

[128] Bai L Constantinides MG Thomas SY Reboulet R Meng F Koentgen F Distinct APCs explain the cytokine bias of α-galactosylceramide variants in vivo. J Immunol (2012) 188:3053–61. 10.4049/jimmunol.1102414 22393151 PMC3311697

[129] McCarthy C Shepherd D Fleire S Stronge VS Koch M Illarionov PA The length of lipids bound to human CD1d molecules modulates the affinity of NKT cell TCR and the threshold of NKT cell activation. J Exp Med (2007) 204:1131–44. 10.1084/jem.20062342 17485514 PMC2118584

[130] van den Elzen P Garg S León L Brigl M Leadbetter EA Gumperz JE Apolipoprotein-mediated pathways of lipid antigen presentation. Nature (2005) 437:906–10. 10.1038/nature04001 16208376

[131] Freigang S Zadorozhny V McKinney MK Krebs P Herro R Pawlak J Fatty acid amide hydrolase shapes NKT cell responses by influencing the serum transport of lipid antigen in mice. J Clin Invest (2010) 120:1873–84. 10.1172/JCI40451 20484813 PMC2877940

[132] Freigang S Landais E Zadorozhny V Kain L Yoshida K Liu Y Scavenger receptors target glycolipids for natural killer T cell activation. J Clin Invest (2012) 122:3943–54. 10.1172/JCI62267 23064364 PMC3484437

[133] Freigang S Kain L Teyton L. Transport and uptake of immunogenic lipids. Mol Immunol (2013) 55:179–81. 10.1016/j.molimm.2012.10.016 23174352 PMC3612381

[134] Prigozy TI Naidenko O Qasba P Elewaut D Brossay L Khurana A Glycolipid antigen processing for presentation by CD1d molecules. Science (2001) 291:664–7. 10.1126/science.291.5504.664 11158680

[135] Winau F Schwierzeck V Hurwitz R Remmel N Sieling PA Modlin RL Saposin C is required for lipid presentation by human CD1b. Nat Immunol (2004) 5:169–74. 10.1038/ni1035 14716313

[136] la Salle de H Mariotti S Angenieux C Gilleron M Garcia-Alles L-F Malm D Assistance of microbial glycolipid antigen processing by CD1e. Science (2005) 310:1321–4. 10.1126/science.1115301 16311334

[137] Miyamoto K Miyake S Yamamura T. A synthetic glycolipid prevents autoimmune encephalomyelitis by inducing TH2 bias of natural killer T cells. Nature (2001) 413:531–4. 10.1038/35097097 11586362

[138] Yu KO Im JS Molano A Dutronc Y Illarionov PA Forestier C Modulation of CD1d-restricted NKT cell responses by using N-acyl variants of alpha-galactosylceramides. Proc Natl Acad Sci U S A (2005) 102:3383–8. 10.1073/pnas.0407488102 15722411 PMC552918

[139] Im JS Arora P Bricard G Molano A Venkataswamy MM Baine I Kinetics and cellular site of glycolipid loading control the outcome of natural killer T cell activation. Immunity (2009) 30:888–98. 10.1016/j.immuni.2009.03.022 19538930 PMC2719696

[140] Bai L Sagiv Y Liu Y Freigang S Yu KO Teyton L Lysosomal recycling terminates CD1d-mediated presentation of short and polyunsaturated variants of the NKT cell lipid antigen alphaGalCer. Proc Natl Acad Sci U S A (2009) 106:10254–9. 10.1073/pnas.0901228106 19506241 PMC2693181

[141] Buatois V Baillet M Bécart S Mooney N Leserman L Machy P. MHC class II-peptide complexes in dendritic cell lipid microdomains initiate the CD4 Th1 phenotype. J Immunol (2003) 171:5812–9. 10.4049/jimmunol.171.11.5812 14634090

[142] Lee YJ Wang H Starrett GJ Phuong V Jameson SC Hogquist KA. Tissue-specific distribution of iNKT cells impacts their cytokine response. Immunity (2015) 43:566–78. 10.1016/j.immuni.2015.06.025 26362265 PMC4575275

[143] Fujii S-I Shimizu K Okamoto Y Kunii N Nakayama T Motohashi S NKT cells as an ideal anti-tumor immunotherapeutic. Front Immunol (2013) 4:409. 10.3389/fimmu.2013.00409 24348476 PMC3845015

[144] Pardoll DM. The blockade of immune checkpoints in cancer immunotherapy. Nat Rev Cancer (2012) 12:252–64. 10.1038/nrc3239 22437870 PMC4856023

[145] Harada Y Imataki O Heike Y Kawai H Shimosaka A Mori S Expansion of alpha-galactosylceramide-stimulated Valpha24+ NKT cells cultured in the absence of animal materials. J Immunother (2005) 28:314–21. 10.1097/01.cji.0000163593.66910.ad 16000949

[146] Giaccone G Punt CJ Ando Y Ruijter R Nishi N Peters M A phase I study of the natural killer T-cell ligand alpha-galactosylceramide (KRN7000) in patients with solid tumors. Clin Cancer Res (2002) 8:3702–9. 12473579

[147] Parekh VV Wilson MT Olivares-Villagómez D Singh AK Wu L Wang C-R Glycolipid antigen induces long-term natural killer T cell anergy in mice. J Clin Invest (2005) 115:2572–83. 10.1172/JCI24762 16138194 PMC1193878

[148] Sullivan BA Kronenberg M. Activation or anergy: NKT cells are stunned by alpha-galactosylceramide. J Clin Invest (2005) 115:2328–9. 10.1172/JCI26297 16138189 PMC1193891

[149] Parekh VV Lalani S Kim S Halder R Azuma M Yagita H PD-1/PD-L blockade prevents anergy induction and enhances the anti-tumor activities of glycolipid-activated invariant NKT cells. J Immunol (2009) 182:2816–26. 10.4049/jimmunol.0803648 19234176 PMC2709814

[150] Sag D Krause P Hedrick CC Kronenberg M Wingender G. IL-10-producing NKT10 cells are a distinct regulatory invariant NKT cell subset. J Clin Invest (2014) 124:3725–40. 10.1172/JCI72308 25061873 PMC4151203

[151] van der Vliet HJ Molling JW Nishi N Masterson AJ Kölgen W Porcelli SA Polarization of Valpha24+ Vbeta11+ natural killer T cells of healthy volunteers and cancer patients using alpha-galactosylceramide-loaded and environmentally instructed dendritic cells. Cancer Res (2003) 63:4101–6. 12874013

[152] Toura I Kawano T Akutsu Y Nakayama T Ochiai T Taniguchi M. Cutting edge: inhibition of experimental tumor metastasis by dendritic cells pulsed with alpha-galactosylceramide. J Immunol (1999) 163:2387–91. 10452972

[153] Chang DH Osman K Connolly J Kukreja A Krasovsky J Pack M Sustained expansion of NKT cells and antigen-specific T cells after injection of alpha-galactosyl-ceramide loaded mature dendritic cells in cancer patients. J Exp Med (2005) 201:1503–17. 10.1084/jem.20042592 15867097 PMC1389847

[154] Nieda M Okai M Tazbirkova A Lin H Yamaura A Ide K Therapeutic activation of Valpha24+Vbeta11+ NKT cells in human subjects results in highly coordinated secondary activation of acquired and innate immunity. Blood (2004) 103:383–9. 10.1182/blood-2003-04-1155 14512316

[155] Kunii N Horiguchi S Motohashi S Yamamoto H Ueno N Yamamoto S Combination therapy of in vitro-expanded natural killer T cells and alpha-galactosylceramide-pulsed antigen-presenting cells in patients with recurrent head and neck carcinoma. Cancer Sci (2009) 100:1092–8. 10.1111/j.1349-7006.2009.01135.x 19302288 PMC11158111

[156] Yamasaki K Horiguchi S Kurosaki M Kunii N Nagato K Hanaoka H Induction of NKT cell-specific immune responses in cancer tissues after NKT cell-targeted adoptive immunotherapy. Clin Immunol (2011) 138:255–65. 10.1016/j.clim.2010.11.014 21185787

[157] Motohashi S Nagato K Kunii N Yamamoto H Yamasaki K Okita K A phase I-II study of alpha-galactosylceramide-pulsed IL-2/GM-CSF-cultured peripheral blood mononuclear cells in patients with advanced and recurrent non-small cell lung cancer. J Immunol (2009) 182:2492–501. 10.4049/jimmunol.0800126 19201905

[158] Okita K Motohashi S Shinnakasu R Nagato K Yamasaki K Sato Y A set of genes associated with the interferon-γ response of lung cancer patients undergoing α-galactosylceramide-pulsed dendritic cell therapy. Cancer Sci (2010) 101:2333–40. 10.1111/j.1349-7006.2010.01696.x 20804502 PMC11159413

[159] Barral P Polzella P Bruckbauer A van Rooijen N Besra GS Cerundolo V CD169(+) macrophages present lipid antigens to mediate early activation of iNKT cells in lymph nodes. Nat Immunol (2010) 11:303–12. 10.1038/ni.1853 20228797 PMC2923071

[160] Macho-Fernandez E Chang J Fontaine J Bialecki E Rodriguez F Werkmeister E Activation of invariant natural killer T lymphocytes in response to the α-galactosylceramide analogue KRN7000 encapsulated in PLGA-based nanoparticles and microparticles. Int J Pharm (2012) 423:45–54. 10.1016/j.ijpharm.2011.04.068 21575695

[161] Nakamura T Yamazaki D Yamauchi J Harashima H. The nanoparticulation by octaarginine-modified liposome improves α-galactosylceramide-mediated antitumor therapy via systemic administration. J Control Release (2013) 171:216–24. 10.1016/j.jconrel.2013.07.004 23860186

[162] Fujii S Goto A Shimizu K. Antigen mRNA-transfected, allogeneic fibroblasts loaded with NKT-cell ligand confer antitumor immunity. Blood (2009) 113:4262–72. 10.1182/blood-2008-08-176446 19164596

[163] Watarai H Fujii S Yamada D Rybouchkin A Sakata S Nagata Y Murine induced pluripotent stem cells can be derived from and differentiate into natural killer T cells. J Clin Invest (2010) 120:2610–8. 10.1172/JCI42027 20516640 PMC2898602

[164] Heczey A Liu D Tian G Courtney AN Wei J Marinova E Invariant NKT cells with chimeric antigen receptor provide a novel platform for safe and effective cancer immunotherapy. Blood (2014) 124:2824–33. 10.1182/blood-2013-11-541235 25049283 PMC4215313

[165] Tian G Courtney AN Jena B Heczey A Liu D Marinova E CD62L+ NKT cells have prolonged persistence and antitumor activity in vivo. J Clin Invest (2016) 126:2341–55. 10.1172/JCI83476 27183388 PMC4887157

[166] Benlagha K Wei DG Veiga J Teyton L Bendelac A. Characterization of the early stages of thymic NKT cell development. J Exp Med (2005) 202:485–92. 10.1084/jem.20050456 16087715 PMC2212852

